# Key Strategies for Optimizing Pediatric Perioperative Nutrition—Insight from a Multidisciplinary Expert Panel

**DOI:** 10.3390/nu15051270

**Published:** 2023-03-03

**Authors:** Mehul V. Raval, Megan A. Brockel, Sanja Kolaček, Kathleen E. Simpson, Elizabeth Spoede, Kathryn N. Porter Starr, Karyn L. Wulf

**Affiliations:** 1 Division of Pediatric Surgery, Department of Surgery, Northwestern University Feinberg School of Medicine, 225 E. Chicago Avenue, Box 63, Chicago, IL 60611, USA; 2 Department of Anesthesiology, Children’s Hospital Colorado, Aurora, CO 80045, USA; 3 Referral Centre for Pediatric Gastroenterology and Nutrition, University Children’s Hospital Zagreb, 10000 Zagreb, Croatia; 4 Department of Pediatrics, University of Colorado, Denver, CO 80204, USA; 5 Pediatric Clinical Dietitian, Texas Children’s Hospital, Houston, TX 77030, USA; 6 Division of Geriatrics, Department of Medicine, Duke University School of Medicine, Durham, NC 27708, USA; 7 Durham VA Health Care System, Durham, NC 27705, USA; 8 Abbott Nutrition, Abbott Laboratories, Columbus, OH 43219, USA

**Keywords:** nutritional status, nutritional assessment, nutrition therapy

## Abstract

Adequate nutrition is an essential factor in healing and immune support in pediatric patients undergoing surgery, but its importance in this setting is not consistently recognized. Standardized institutional nutrition protocols are rarely available, and some clinicians may be unaware of the importance of assessing and optimizing nutritional status. Moreover, some clinicians may be unaware of updated recommendations that call for limited perioperative fasting. Enhanced recovery protocols have been used in adult patients undergoing surgery to ensure consistent attention to nutrition and other support strategies in adult patients before and after surgery, and these are now under evaluation for use in pediatric patients as well. To support better adoption of ideal nutrition delivery, a multidisciplinary panel of experts in the fields of pediatric anesthesiology, surgery, gastroenterology, cardiology, nutrition, and research have gathered and reviewed current evidence and best practices to support nutrition goals in this setting.

## 1. Introduction

More than 3.9 million operations are performed on children in US hospitals every year, which represents nearly 5% of the pediatric population [1]. Although overall perioperative and postoperative mortality rates are low in children [2], surgery is a common cause of morbidity worldwide [3]. An anabolic state is required for optimal clinical outcomes and healing, yet the role of nutrition is still underestimated and not consistently recognized as an integral tool to address catabolism and, therefore, to promote necessary healing and immune support, especially in pediatric surgical recovery. Available evidence suggests that impaired nutritional status in pediatric patients undergoing surgery increases the risks of infectious and other postoperative complications [2,4,5], prolongs length of hospital stay [4], increases costs of care [6], and increases mortality risk [2].

Adequate nutritional status is critical through all phases of patient care, from the preoperative period through the postoperative recovery period. The use of enhanced recovery protocols—evidence-based clinical strategies to increase the speed of recovery and that emphasize the importance of nutrition—have been successful in adult populations [7,8,9,10,11] and are now being evaluated for their potential utility in pediatric surgery populations [12,13,14,15,16,17].

Although clinicians may recognize the role nutrition plays across the spectrum of pediatric surgical planning and recovery, standardized institutional nutrition protocols are rarely available and may be inconsistently applied. Some clinicians may be unaware of the importance of assessing and, if needed, optimizing nutritional status in the preoperative setting, especially with respect to updated recommendations to limit perioperative fasting. Achieving a cohesive and effective nutrition strategy in these patients is especially complex due to the number of clinician disciplines included in care decisions.

To increase awareness and education in this area, and to reflect the interprofessional disciplines involved in patient care, an expert panel representing the fields of pediatric anesthesiology, surgery, gastroenterology, cardiology, nutrition, and research was convened in November 2021 to discuss important aspects of nutritional support from the early preoperative planning period through late postoperative recovery. Each expert presented on a topic to support further discussion on key issues specific to nutrition in this setting. Topics for discussion included: practical aspects of surgical planning and prehabilitation; emerging enhanced recovery protocols for pediatric populations; specific nutrition recommendations from adult populations that may be applied in pediatric patients undergoing surgery; and special perioperative nutrition considerations in pediatric patients undergoing cardiovascular, neuromuscular, or spinal surgery. Because the topic of perioperative nutrition in patients undergoing surgery continues to evolve and robust evidence is somewhat limited, we prepared for our discussion and subsequent collaboration on this topic with a review of available literature to support and add context to our evaluation of current best practices for nutritional support of pediatric patients undergoing surgery. In this resulting document, we consider available evidence and provide an overview of specific nutritional interventions in pediatric surgical patients that is summarized in a streamlined plan for optimal nutritional care in this setting.

## 2. The Importance of Preoperative Nutrition Screening and Prehabilitation

Adequate preoperative nutrition screening and support are vital yet underrecognized aspects of ensuring optimal surgical outcomes, often due to an inconsistent use of available screening tools in the inpatient pediatric setting, and a lack of validated screening tools in the outpatient setting. Overall, screening in pediatric patients undergoing surgery to identify individuals with malnutrition or those at risk of malnutrition is underrecognized but nevertheless important. Malnutrition is more prevalent in pediatric patients with underlying chronic medical conditions but can be present and underrecognized in the general pediatric population as well. Screening may identify both patients who are underweight and malnourished, and those who are malnourished with obesity [5]. Preexisting malnutrition in pediatric surgical patients results in similar adverse outcomes to critically ill pediatric counterparts [18], and chronic undernutrition also increases the risk of hospital-acquired infections [19]. Overall, the poor outcomes associated with inadequate preoperative nutrition have consequences for health care costs as well [6]. In contrast to assessing anthropometric measures, the main goal of this screening is to assess malnutrition risk and thereby prevent malnutrition, and to identify patients in need of further nutritional assessment.

### 2.1. Strategies for Preoperative Nutrition Screening

There is often limited availability of validated screening tools that clinicians can use to perform adequate preoperative nutrition screening in the outpatient setting and to identify patients requiring additional support with enough planning time before surgery. A summary of several screening tools for malnutrition is available in Table 1. For instance, the subjective global nutritional assessment (SGNA) is routinely used to identify malnutrition risk in pediatric populations [20,21], but is not specifically designed for pediatric patients anticipating surgery.

One study evaluated the relative value of available screening tools for identifying malnutrition at the time of hospital admission in pediatric patients. These included the Screening Tool for Risk on Nutritional Status and Growth (STRONGkids) and the pediatric nutrition screening tool (PNST) administered by nursing staff, with the SGNA administered by a dietitian as a reference control. Neither tool was found to be highly consistent with the SGNA, but the PNST with adjusted cutoffs was found to be appropriate in this setting [22]. Another cross-sectional study compared the reliability and sensitivity of STRONGkids, PNST, and the Pediatric Yorkhill Malnutrition Score (PYMS) in 176 children ages 1–16 years admitted to a second-line hospital. The PYMS exhibited high rates of sensitivity for weight for age (WFA) and body mass for age (BFA) (90.9% and 84.6%, respectively). The PNST tool, meanwhile, performed at a sensitivity rate of 88.9% for height for age (HFA). The researchers concluded that both the PYMS and PNST are suitable tools in the pediatric inpatient setting for malnutrition risk assessment [23]. The PNST with modified criteria for preoperative patients may, therefore, be suggested as an alternative to the SGNA, especially as this tool may be more easily implemented in a wider variety of preoperative patient settings.

Some children may present with medical conditions or syndromes that make established screening tools unreliable. Obtaining basic anthropometrics like weight, height, and BMI, may be difficult in these patients and may skew screening tools. Other anthropometric measures such as mid-upper arm circumference (MUAC) have been proposed as important tools to identify nutrition risk and help determine nutritional status in children with complex underlying conditions. Meanwhile, some children may present with underlying medical conditions or genetic syndromes that make anthropometric measurements unreliable and, therefore, requires a clinician skilled in SGNA to assess nutritional status. Regardless of the screening tool or anthropometric measures used in the preoperative setting, children found to have potential nutrition deficits should be further assessed for potential interventions to strengthen their nutritional status as appropriate. Patients who are found without malnutrition should nevertheless be encouraged to maintain a healthy diet prior to surgery.

The goal of nutritional support in patients identified as at risk for, or having, malnutrition is to improve nutrient stores. However, it is important to understand that achieving this goal could more realistically require weeks or months of planning and assistance, which may not be feasible prior to surgery [24]. For instance, patients who are overweight or obese should not be encouraged to lose weight before the procedure, as this may induce a catabolic state when an anabolic state is ideal in patients preparing for surgery to support the healing and immune support required for successful recovery. A screening tool has no utility in these patients and anthropometric measures and plotting suffice. Patients who are overweight or obese should also be encouraged to focus on healthy eating habits that provide high-quality protein to support muscle mass and a nutrient dense diet to ensure quality nutrient stores, as well as adequate hydration. Any weight loss efforts should be made after the postoperative recovery period, with an emphasis on individual patient goals to motivate behavior change.

### 2.2. Nutritional Support as Part of a Larger Prehabilitation Strategy

Effective pediatric surgical management increasingly recognizes the importance of prehabilitation, which is the practice of supporting patients with a variety of multidisciplinary preoperative interventions—including physical activity, physical therapy, and nutrition—to improve postoperative outcomes. Effective prehabilitation, particularly in adults, begins with the goal of increasing functional capacity at least 4 weeks prior to the procedure to improve postsurgical outcomes [25]. Enhanced recovery protocols include prehabilitation strategies to ensure proper perioperative support, with an emphasis on adequate nutrition. Initial research suggests that enhanced recovery protocols improve outcomes for critically ill pediatric patients [18] and confirm similar findings in adult patient populations [7,8,9,10,11,12,24]. Nutrition prehabilitation is increasingly seen as crucial for patients planning for surgery [26]. As previously mentioned, although current nutrition prehabilitation strategies usually provide intervention at least 10 to 14 days before surgery, achieving consistently better nutrient stores could take a longer planning and support period that may not be feasible [24]. For practical purposes, an enhanced recovery protocol checklist for hospital staff, as well as department-specific enhanced recovery champions, may also improve compliance across treatment settings. A generic protocol to guide implementation of enhanced recovery care throughout all phases of perioperative care is presented in Table 2.

## 3. Perioperative Nutrition

Although both adult and pediatric patients had been discouraged from nutritional intake close to surgery since at least the 19th century [27], clinicians now recognize that the cessation of nutrition is not necessary and adequate nutrition through this period can support improved outcomes. Current guidelines recognize the need to avoid prolonged fasting [28,29,30,31], but clinical practice has been slow to adopt this mindset due to entrenched dogma and surgical scheduling limitations that remain barriers to implementation. A summary of available guidelines on feeding in preoperative, perioperative, and perioperative settings is presented in Table 3. Note that fasting guidelines differ in the US (clear liquids allowed up to 2 h prior to surgery) and Europe (typically 1 h prior to surgery).

In adult patients, long periods of fasting may result in insulin resistance, rebound hyperglycemia, the need for exogenous insulin, and a failure to achieve an anabolic state [32]. Enhanced recovery strategies in adults that include adequate nutrition have demonstrated improved insulin sensitivity [33], and clear liquid intake until 2 to 4 h before general anesthesia has likewise been associated with better postoperative outcomes [28,34].

The benefits of minimal fasting may extend to pediatrics, and only 1 to 2 h of preoperative fasting (depending on European or US guidelines, respectively) is required to minimize aspiration risk in most patients, understanding that specific conditions such as gastroesophageal reflux disease (GERD) or gastritis may need a longer fasting time before surgery [35]. In a recent analysis of infants undergoing surgery for cyanotic congenital heart disease, a 1-h fast resulted in lower rates of caregiver-reported and clinical effects such as crying, thirst, and hypoxia compared with those undergoing a 2-h preoperative fast, without increasing aspiration risk [36]. Current guidelines suggest that prolonged fasting in pediatric surgical patients unnecessarily interrupts the delivery of optimal enteral nutrition after surgery [31]. Despite concerns about delayed gastric emptying with surgery, current evidence suggests that measures used to assess delayed gastric emptying such as gastric residual volume (GRV) are inaccurate and do not reflect the risk of aspiration and the ability to rapidly resume enteral feeding [31]. Adequate fluids before surgery may be especially important to hydration and reduce the need for intraoperative intravenous fluids [35]. Proper patient assessment is required to reduce the risk of dehydration by ensuring that adequate fluids are consumed and maintained prior to the surgical procedure [35]. Reduced fasting time may also impact subjective measures of improved outcomes, including reduced crying and anxiety [35,37].

### 3.1. Evidence for Preoperative Carbohydrate Loading

In adult populations, oral carbohydrate loading before surgery has been investigated as a strategy to improve outcomes. Multiple studies have reported a reduced length of stay and increased postoperative insulin sensitivity [38,39,40,41,42,43]. Oral carbohydrate loading has also been associated with reduced thirst, need for ionotropic support, and incidence of arrythmias, anxiety, hunger, nausea, vomiting, and pain [38,40,41,42,43]. Meanwhile, high-quality evidence is still lacking in specific adult patient populations, such as those with diabetes [44,45,46].

In pediatrics, current evidence is limited but suggests that limited fasting and preoperative carbohydrate delivery in pediatric patients improves outcomes. In randomized, controlled trials of pediatric patients undergoing upper gastrointestinal endoscopy while under general anesthesia, children who received a carbohydrate beverage before the procedure exhibited lower rates of nausea and gastric contents than those who underwent a standard period of fasting, suggesting that this strategy did not increase aspiration risk under anesthesia [47]. There is a need for additional research to answer important clinical questions on carbohydrate loading, especially with respect to its relative value in specific pediatric and adolescent age groups, different surgical populations, and any changes required in patients with certain conditions such as diabetes. Ideal dosing and timing strategies also remain unclear in pediatric patients. As evidence continues to accumulate in adult patients, future attention should be paid to better characterizing these perioperative feeding strategies in pediatric patients.

### 3.2. Strategies to Improve Perioperative Nutrition Delivery

Both clinicians and caregivers may continue to demonstrate hesitance in providing recommended nutrition in the hours leading up to surgery. Indeed, while extended preoperative fasting is no longer recommended, many patients are still instructed to fast for much longer periods that what is clinically indicated, suggesting additional clinician education is needed to appropriately guide patients and caregivers [48]. To improve nutrition intake closer to surgery, especially before hospital arrival, targeted educational materials for patients and caregivers may be helpful to emphasize the value of preoperative clear fluids. Finally, recommending specific amounts of fluids in addition to timing before surgery may increase compliance.

### 3.3. Early Oral and Enteral Nutrition during the Postoperative Period

Nutrients in the gut are necessary to strengthen the mucosal barrier, which may also contribute to maintaining enteral nutrition. Early oral or enteral feeding initiated within the 24 h after surgery has been shown to improve outcomes in adult populations, resulting in lower rates of complications and mortality and shorter length of stay [49,50,51,52]. Early enteral nutrition has also demonstrated value in pediatric surgery settings, including gastrointestinal surgery, with decreased length of stay and risk of complications and infections [53,54,55]. In pediatric patients, improvements have been noted not only in early postoperative outcomes but also in later functional outcomes [53,54,55,56].

The introduction of intake and mode of feeding may need to be adjusted based on the surgical type and severity of underlying disease. However, evidence suggests that in order to maintain gut function, it is critical to provide minimal amounts of enteral nutrition even if additional parenteral support is required. One systematic review and meta-analysis found enteral nutrition to be preferable to parenteral nutrition after gastrointestinal surgery [57], and researchers have proposed that gut-derived sepsis may play a role in adverse outcomes when its function is not supported with continued enteral nutrition [58,59].

### 3.4. Practical Strategies to “Advance Diet as Tolerated”

Strategies to plan and advance an enteral diet ideally should begin in the perioperative period so that it may be sustained well into the postoperative and recovery settings. Current evidence suggests that nutritional status be optimized for at least 2 weeks after surgery and that extended monitoring is also warranted, especially in patients with malnutrition [60]. Practical stepwise progression of diet advancement with educational handouts/infographics could be helpful for patients and caregivers to prevent over-ambitious oral consumption post-surgery. Overall, a consistent oral diet should be established that provides high-quality nutrition while avoiding overconsumption pre- and post-procedure.

## 4. Post-Discharge Nutrition Planning

Ongoing adequate nutrition intake is an essential aspect of postsurgical recovery but may be overlooked without adequate understanding of the role that nutrition plays in postoperative recovery and a long-term return of adequate functional status. Nutritional goals in the early postoperative period should focus on a return or maintenance of an anabolic state that achieves healing and net muscle gain. In subsequent months after surgery, nutritional status should be serially reassessed, especially in patients identified as malnourished before or following surgery that require catch-up growth.

Dietitian support should be sought for patients who continue to struggle with weight issues, including inadequate weight gain, inability to achieve catch-up growth, overweight/obesity, and more specific concerns about ongoing nutritional deficits. Patients and caregivers also play a central role in maintaining optimal nutritional status after surgery. To facilitate their participation, patients and caregivers should receive education and supplemental materials before surgery discussing how to optimize nutrition prior to surgery and different feeding options that may be encountered in the hospital, with reminders and follow-up support given in the recovery period. Ongoing nutrition instructions should always be included in discharge planning, with patients and caregivers present when nutrition goals are established. These nutrition recommendations should also be communicated to the larger care team, including the outpatient clinicians with whom the child will follow up. The location and timing of the provision of nutrition recommendations and counseling may depend on individual patient factors, especially considering the ongoing COVID-19 pandemic. Nevertheless, clinicians, dietitians, patients, and caregivers can continue to identify flexible strategies for ongoing nutrition support, including telemedicine options and integration into routine care appointments.

## 5. Educational Opportunities to Advance Nutritional Support

As nutritional recommendations from the preoperative setting through recovery continue to evolve, additional education for clinicians is needed for adequate nutrition in pediatric surgery. As previously discussed, nutrition interventions as part of an enhanced recovery strategy have the potential to improve outcomes in both adult [7,8,9,10,11] and pediatric [12,14,15,16,17] surgical patients. Education should focus on the practical implementation of established, enhanced recovery protocols [61,62] and strategies to tailor these protocols to specific institutions, care settings, and patient populations.

One of the most significant barriers to adequate nutrition is a lack of understanding among clinical staff, patients, and caregivers of the importance of nutrition in pediatric surgery, coupled with a reliance on outdated paradigms of perioperative fasting. Practitioners who are not routinely involved in perioperative planning may require additional education on this topic from surgical planning through recovery. Specific messaging can be tailored to each stakeholder group that plays a role in nutrition planning and ongoing support, as summarized in Table 4.

## 6. Opportunities for Further Research

Although we have repeatedly emphasized the importance of screening, available screening tools are not specific to the pediatric surgery population [20,21]. Identifying children at nutritional risk and intervening appropriately has demonstrated the ability to improve postoperative outcomes [24,63], and future research may identify assessment tools specific to surgery that can be widely implemented to identify patients at risk and in need of additional intervention.

Likewise, it is clear from current clinical practice and limited available guidance that more specific recommendations to minimize perioperative fasting would be helpful. Clinicians, patients, and caregivers may be more likely to follow recommendations of limited preoperative fasting and early nutrition provision after surgery if supported by a greater weight of evidence and stronger guidance. Strategies such as carbohydrate loading before surgery also deserve additional research attention, especially in pediatric surgery patients, with a focus on age groups most likely to benefit, ideal dosing and timing, and effects of health conditions, such as diabetes, that impact its potential value. Early enteral feeding after surgery, especially as a strategy to maintain gut function, is another area for additional research that could support consistent practices in pediatric surgery.

## 7. Discussion

Based on this expert panel review of available literature, five key areas of focus were identified related to perioperative nutrition for children undergoing surgery. The first point is that adequate preoperative nutrition screening and support are vital yet underrecognized aspects of ensuring optimal surgical outcomes, and therefore support for all pediatric patients undergoing surgery should include care based on concepts of prehabilitation and enhanced recovery. Second, current guidelines limit preoperative fasting to 1 to 2 h, and patients should be educated on fasting guidelines before surgery to avoid dehydration. Preoperative carbohydrate loading should also be considered. The third point is that after surgery, ongoing nutrition provision is an important aspect of recovery but may be overlooked without an adequate understanding of the role that nutrition plays in postoperative recovery and a long-term return of adequate functional status. Fourth, all clinical staff responsible for patient care, patients, caregivers, and hospital administration require tailored education that emphasizes the importance of nutrition in this setting. Finally, research is needed to develop screening tools that are specific to the pediatric surgery population, clear recommendations to minimize perioperative fasting, and additional weight of evidence for strategies such as prehabilitation, carbohydrate loading before surgery, and early enteral feeding after surgery. More pragmatic and prospective data are needed to evaluate successful implementation of recommendations and to better understand clinical outcomes as well as patient/family-reported outcomes. Multicenter collaborative efforts would be beneficial to explore contextual adaptations that would aid generalizability and acceptance of practice changes in diverse practice settings.

Limitations to our review of this topic should be highlighted, especially the fact that our review is based on our brief 1-day discussion and a review of available literature. These recommendations are not consistently based on prospective data in these patients and are often extrapolated from findings in adult patients undergoing surgery. The applicability of our findings should therefore be considered with caution, but we have identified key areas for future research, namely the identification of reliable malnutrition screening tools, and the impact of specific fasting and feeding protocols on patient outcomes.

## 8. Conclusions

Malnutrition increases the risk of complications, length of stay, and costs of care in pediatric patients undergoing surgery. Even those without previous nutritional challenges may face an increased risk of malnutrition perioperatively or during recovery. Nutritional assessments and interventions play an important role in the outcomes of a variety of pediatric surgical patients. These should be included as part of broader enhanced recovery discussions and protocols with input and ownership from all stakeholders, including patients, caregivers, primary care providers, subspecialty clinicians, surgeons, anesthesiologists, nurses, dietitians, and hospital administration. In pediatric surgery, the ultimate nutritional goal is to optimize nutrition preoperatively, and sustain nutrition provision after surgery and through recovery while also improving patient outcomes. A focus on the key strategies discussed, as well as future research that clarifies ideal malnutrition screening tools, limited preoperative fasting, and early postoperative nutrition in pediatric surgery should help achieve improved postoperative outcomes while promoting the long-term goal of adequate growth and physical function.

## Figures and Tables

**Table 1:** Potential Nutrition Screening Tools Applicable to Pediatric Patients Undergoing Surgery.

Screening Tool	Attributes/Benefits	Limitations	Validation
Subjective global nutritional assessment (SGNA) [20,21]	Gold standard screening tool	Best performed by a dietitian	Not validated for pediatric surgery
Screening Tool for Risk on Nutritional Status and Growth (STRONGkids) [22]	Quick, straightforward	May overestimate nutrition risk	Validated for hospitalized pediatric patients
Pediatric nutrition screening tool (PNST) [22]	With modified criteria, may be implemented across a variety of patient settings	May underestimate nutrition risk	Validated for hospitalized pediatric patients
Mid-upper arm circumference (MUAC)	Used in complex cases when obtaining measures of weight, height, and BMI are difficult	User training necessary, not consistently used by clinicians	Not validated for pediatric surgery

**Table 2:** Important Nutritional Aspects of an Enhanced Recovery Protocol.

	Description	Target Stakeholder
Preoperative	Education on nutrition importance and agreement on a nutrition care plan	Patients/caregivers Primary care providers
	Nutrition screening to assess malnutrition risk	Patients/caregivers Dietitians
	Preoperative nutritional assessment/intervention as needed	Patients/caregivers Dietitians Primary care providers
	Secure adequate access to high-quality protein and nutrition-dense dietary components both before and after surgery	Patients/caregivers Social workers/Dietitians
	Establish a plan for carbohydrate loading	Patients/caregivers Primary care providers Subspecialty clinicians
Perioperative	Clear instructions for limited fasting (1–2 h) ^1^ and carbohydrate loading	Patients/caregivers Surgeons/anesthesiologists Nurses Hospital administration
	Establish a plan for early advancement of oral or enteral feeding after surgery	Patients/caregivers Subspecialty clinicians Dietitians Hospital administration
Postoperative	Establish a strategy for ongoing nutritional assessments and support after surgery	Patients/caregivers Specialty clinicians Primary care providers Dietitians

^1^ Guidelines vary by country.

**Table 3:** Current Guideline Recommendations on Preoperative Fasting in Pediatric Patients Undergoing Surgery.

Guideline	Preoperative	Perioperative and Postoperative
American Society of Anesthesiologists Task Force on Preoperative Fasting and the Use of Pharmacologic Agents to Reduce the Risk of Pulmonary Aspiration [28]	For otherwise healthy infants, children, and adults “clear liquids may be ingested for up to 2 h before procedures requiring general anesthesia, regional anesthesia, or procedural sedation and analgesia” (up to 4 h before procedures for breast milk, up to 6 h before procedures for infant formula)	
European Society of Anaesthesiology [29]	“It is safe and recommended for all children able to take clear fluids, to be allowed and encouraged to have them up to 1 h before elective general anaesthesia” Type and volume of fluids might be specified or defined by national scientific societies and/or local institutional policies	
European Society of Anaesthesiology and Intensive Care (ESAIC) [30]	Minimum preoperative fasting times of 6 h for solids, 4 h for infant formula, 3 h for breast milk, and 1 h for clear fluids (6-4-3-1 strategy)	Encourage early postoperative feeding
Society of Critical Care Medicine and American Society for Parenteral and Enteral Nutrition (SCCM/ASPEN) [31]		Prolonged fasting unnecessarily interrupts the delivery of optimal enteral nutrition after surgery Despite concerns about delaying gastric emptying with surgery, measures such as gastric residual volume (GRV) are inaccurate and do not reflect the risk of aspiration and the ability to rapidly resume enteral feeding

**Table 4:** Stakeholder Messaging for Improved Nutritional Assessment and Management in Pediatric Surgery.

Stakeholder Group	Messaging
Patients and Caregivers	Emphasize why nutritional support is important and how to achieve consistent pre-, peri-, and postoperative nutrition; explain their central role in a larger enhanced recovery protocol.
Surgeons and Anesthesiologists	Encourage a pivot from outdated dogma on preoperative fasting with data on costs incurred with surgical rescheduling when outdated NPO guidance is not followed. Encourage clinicians to encourage prompt resumption of enteral intake in the postoperative period as clinically appropriate.
Subspecialty Clinicians	Communicate the importance of routine screening and monitoring of nutritional status throughout the surgical journey from the early preoperative period through recovery.
Primary Care Providers	Focus on helping patients and caregivers understand why perioperative nutrition is important and why enhanced recovery strategies with adequate nutrition after surgery result in better outcomes.
Nurses	Communicate why nutrition is important in this setting and their role in providing adequate support from the preoperative period through recovery.
Dietitians	Emphasize the role of nutrition in enhanced recovery and strategies to support health care professionals, patients, and caregiver education.
Social Workers	Provide strategies for addressing potential food insecurity and identification of assistance programs.
Hospital Administrators	Demonstrate the potential cost implications of inadequate nutritional support and the cost savings of improved outcomes, including reduced length of stay and decreased readmissions.

NPO: nil per os (nothing by mouth).

## Data Availability

Not applicable.
